# Mechanism of the chromosome-induced polar body extrusion in mouse eggs

**DOI:** 10.1186/1747-1028-6-17

**Published:** 2011-08-25

**Authors:** Qiong Wang, Catherine Racowsky, Manqi Deng

**Affiliations:** 1 Department of Obstetrics and Gynecology and Reproductive Biology, 75 Francis Street, Brigham and Women's Hospital, Harvard Medical School, Boston, MA 02115, USA; 2 Center for Reproductive Medicine, Department of Gynecology and Obstetric, First Affiliated Hospital of Sun Yet-Sen University, 58 Zhongshan 2nd Road, Guangzhou, P. R. China

## Abstract

**Background:**

An oocyte undergoes two rounds of asymmetric division to generate a haploid gamete and two small polar bodies designed for apoptosis. Chromosomes play important roles in specifying the asymmetric meiotic divisions in the oocytes but the underlying mechanism is poorly understood.

**Results:**

Chromosomes independently induce spindle formation and cortical actomyosin assembly into special cap and ring structures in the cortex of the oocyte. The spindle and the cortical cap/ring interact to generate mechanical forces, leading to polar body extrusion. Two distinct force-driven membrane changes were observed during 2^nd ^polar body extrusion: a protrusion of the cortical cap and a membrane invagination induced by an anaphase spindle midzone. The cortical cap protrusion and invagination help rotate the spindle perpendicularly so that the spindle midzone can induce bilateral furrows at the shoulder of the protruding cap, leading to an abscission of the polar body. It is interesting to note that while the mitotic spindle midzone induces bilateral furrowing, leading to efficient symmetric division in the zygote, the meiotic spindle midzone induced cytokinetic furrowing only locally.

**Conclusions:**

Distinct forces driving cortical cap protrusion and membrane invagination are involved in spindle rotation and polar body extrusion during meiosis II in mouse oocytes.

## Background

Female meiosis in most animals is characterized by two sequential asymmetric meiotic divisions following one round of DNA replication, which results in formation of a haploid egg and extrusion of two small polar bodies destined for degeneration. Oocyte haploidization by means of discarding half of the chromosomes into the polar bodies represents a special mechanism for female gamete formation. To accomplish asymmetric cell division, a cell needs to establish a cortical polarity, according to which the mitotic/meiotic spindle is asymmetrically positioned [1-3]. The highly asymmetric cell divisions during female meiosis ensure that the produced haploid gametes maximally inherit maternal components, which are beneficial for embryo development.

Although polar body extrusion during female meiosis has been recognized for many years, the mechanism by which oocytes accomplish the special asymmetric divisions is still poorly understood. Early studies have noted that the interaction of the chromosomes and the cortical cytoskeleton plays important roles in polar body extrusion [4,5]. The mechanism of spindle rotation, cytokinesis, particularly the involved mechanical forces for polar body extrusion, are not well understood. Our recent studies have shown that chromosomes induce cortical actin and myosin II assembly into a distinct actin cap surrounded by a myosin II ring in the MII eggs [6]. Interestingly, sperm chromatin incorporation at fertilization or microinjection of DNA beads into MII eggs induce cortical actin cap/myosinII ring similar to that induced by maternal chromosomes [7]. The chromosome-induced cortical actin cap/myosin II ring undergoes protrusion during metaphase-anaphase transition, forming a cone [7,8] but little is known about its role in spindle rotation and polar body extrusion. It is known that during symmetric cell division in mitosis, an anaphase spindle midzone induces bilateral furrowing from the opposite cortex [9,10], which results in bisection of the mother cell into two daughter cells of similar size. However, little is known about the cytokinetic processes during polar body extrusion.

In the present study, we injected DNA coated beads into mouse MII eggs to mimic the chromosomes, and show that the chromosomes induce cortical actomyosin assembly and spindle formation independently, most likely by different chromosome signals. While the chromosome-induced cortical cap undergoes protrusion, the anaphase spindle midzone first induces a unilateral furrow, which coordinates with the cap protrusion to cause spindle rotation. After successful spindle rotation with one spindle pole dragged by the protruding cortical cap and the other spindle pole positioned in the cytoplasm, the spindle midzone induces bilateral furrowing which leads to an abscission of the polar body.

## Results

### Differential induction of cortical actomyosin assembly and spindle formation by the chromosomes

**Figure 1:**
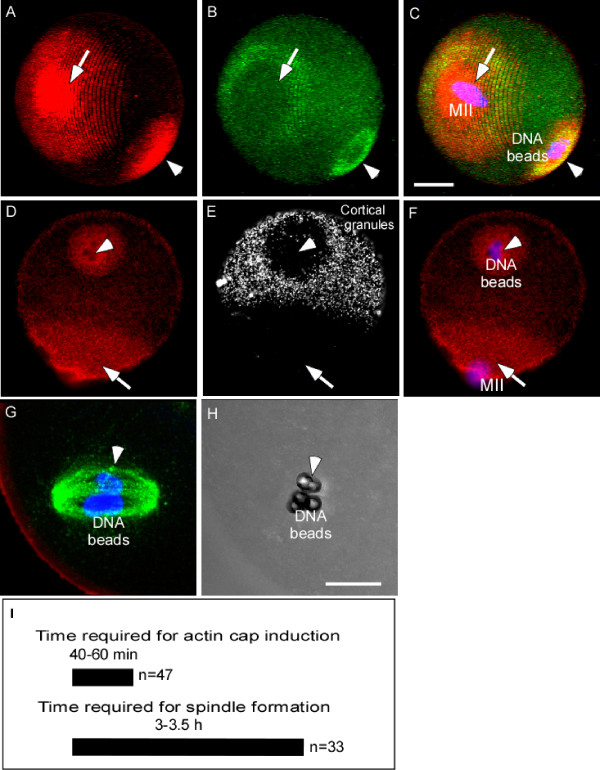
**Differential induction of cortical polarity and spindle formation by chromatin**. (A-C) DNA bead-induced an actin cap (A, red, arrowhead) and a myosin II ring (B, green, arrowhead) which are comparable to those induced by maternal chromosomes (indicated by MII, A-C, arrows). (D-F) DNA bead-induced cortical actin cap (shown in red) and cortical granule (CG) redistribution (shown in white). Note that the injected DNA beads induced formation of a CG free domain (E, arrowhead), which is overlapping with the actin cap (D and E, arrowhead). The arrows point to the MII chromosome region. DNA is shown in blue in all the figures unless otherwise stated. (G) A bipolar spindle induced by the injected DNA beads. In this image, the microtubules are shown in green. (H) A DIC image of G showing the DNA beads. (I) A comparison of the time required for the cortical actin cap and spindle formation induced by DNA beads. The scale bars shown in all of the figures represent 20 μm unless otherwise stated.

It is known that chromosomes are able to induce both microtubule assembly into a bipolar spindle and cortical actomyosin assembly into a cap and ring [6]. It is unclear however, whether the induction of spindle and cortical polarity requires the same chromosome signal. We have shown that DNA beads injected into the cortex of the MII eggs behaved as the in vivo chromosomes, inducing a cortical actomysin cap/ring (Figure 1A-C, arrowheads), formation of a cortical granule (CG) free domain (Figure 1D-F, arrowheads), and a bipolar spindle (Figure 1E, F) comparable to those induced by meiotic chromosomes [6]. The induction of the cortical actin cap and the CG free domain by DNA beads was consistent with sperm chromatin injection as we reported earlier [11]. By injecting DNA beads into the cortex of the MII eggs, we were able to compare the time required for cortical actomyosin assembly and spindle formation when the chromosome signals were positioned in the cortex. It was noted that the induction of cortical actomyosin assembly was faster than that of spindle formation (Figure 1G). In addition, the chromosome-induced actomyosin cap and spindle formation are independent from each other. Our previous results have shown that disruption of spindle formation by nocodazole does not affect the DNA bead-induced cortical cap formation [6] and both meiotic chromosomes and DNA beads can induce spindle formation in the absence of cortical cap induction especially when they were positioned far away from the cortex (data not shown) [6]. It was noted that 23 out of 49 eggs that were cortically injected with DNA beads showed cortical cap formation without spindle formation (data not shown). All these results suggest that the induction of the cortical cap and spindle formation require different chromosomal signals.

### Microinjection of DNA beads revealed sequential assembly of cortical actomyosin cap and myosin II ring

**Figure 2:**
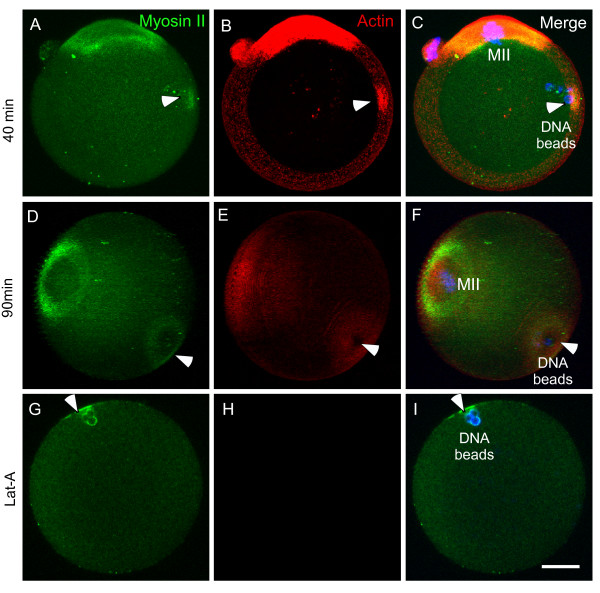
**Sequential induction of cortical cap and myosin II ring by DNA beads**. (A-C) DNA beads first induced a myosin II cap (A, green, arrowhead) and an overlapping actin cap (B, red, arrowhead) at 40 min after microinjection. Shown are representative images of 17 analyzed eggs. (D-F) DNA bead-induced myosin ring formation (D, green, arrowhead) surrounding an actin cap (E, red, arrowhead) observed at 90 min after injection (observation of over 50 eggs). (G-I) DNA bead-induced myosins II cap instead of ring formation (G, green, arrowhead) after disruption of actin by Lat-A. Note in H that actin (red) is not visible after Lat-A treatment.

We further followed up the kinetics of actin and myosin II assemblies that are induced by the injected DNA beads. It was interesting to note that myosin II and actin first formed an overlapping cortical cap (Figure 2A-C, arrowheads). The myosin II-formed cortical cap was later reorganized into a ring surrounding the actin cap (Figure 2D-F, arrowheads), which is comparable to that observed overlying the maternal MII chromosomes (Figure 2D-F). This suggests that actin and myosin II first form a cortical cap, which then reorganizes into a myosin II ring surrounding the actin cap. Disruption of the actin cap by Lat-A had no effect on the DNA bead-induced myosin II cap formation but prevented the subsequent myosin II cap reorganization into a ring (Figure 2G-I). Consistently, the pre-formed myosin II rings changed back to the cap after Lat-A treatment and interestingly, after washing out of Lat-A, the myosin II caps were reorganized into rings again (data not shown). This suggests that the chromosome-induced actomyosin cap and ring are interchangeable structures, and an actin cap is required for both the formation and the maintenance of the myosin II ring.

### The oocyte cortex underwent distinct protrusion and invagination which led to polar body extrusion

**Figure 3:**
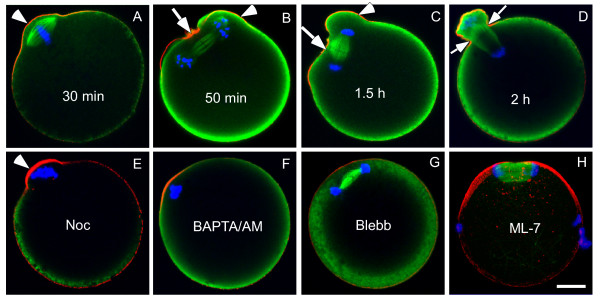
**Cortical protrusion and spindle midzone-induced membrane furrowing during polar body extrusion**. (A-D) Different time points after SrCl_2 _treatment showing the cortical cap protrusion (arrowheads) and the spindle midzone-induced membrane furrowing (arrows). Note the cortical protrusion overlying the chromosomes (arrowheads) and the spindle midzone-induced membrane furrows changing from the initial unilateral (B and C, arrowheads) to the eventual bilateral (D, arrowheads). (E) Cortical cap protrusion (arrowhead) after disruption of spindle microtubules by nocodazole. (F) Block of cortical protrusion by BAPTA/AM. (G, H) Block of cortical protrusion by blebbistatin and ML-7 during egg activation.

**Figure 4:**
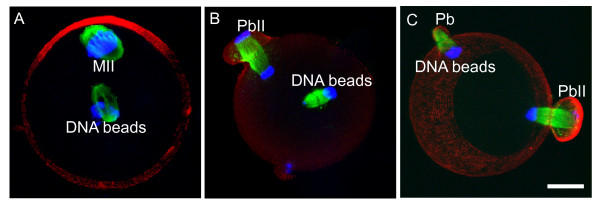
**A distance-dependent membrane furrow induction by the spindle midzone**. (A) DNA bead-induced spindle formation at the center of an MII egg. (B) After inducing anaphase by SrCl_2_, the centrally positioned anaphase spindle was unable to induce membrane furrowing. Note that the cortically positioned maternal chromosomes and spindle (indicated by MII) induced the 2^nd ^polar body extrusion (PbII). (C) The DNA bead-induced spindles were able to induce membrane furrowing and polar body extrusion (indicated by Pb) if positioned close to the cortex.

Meiotic spindles in the MII eggs are usually positioned parallel to the cortex [12]. After egg activation (induced either parthenogenetically by SrCl_2 _or fertilization), the oocytes underwent anaphase onset. It is interesting to note two distinct deformations in the cortex during polar body extrusion: the membrane protrusion from the cortical cap (Figure 3A, arrowhead) and a unilateral membrane furrow overlying the anaphase spindle midzone (Figure 3B, arrow). These cortical changes seem to be coupled with the rotation of the spindle during polar body extrusion. After the spindle is rotated to acquire a perpendicular position, bilateral furrows were observed at the shoulder of the protruding cap (Figure 3C, D), which led to an abscission of the polar body (Figure 4D arrows, Additional File 1).

Disruption of spindle microtubules by nocodazole had no effect on the above described cortical cap protrusion (Figure 3E), suggesting that the process is independent of the spindle. It is known that fertilization or parthenogenetic activation of eggs by SrCl_2 _induces oscillatory changes of intracellular Ca^2+ ^concentration [13,14]. To determine whether Ca^2+ ^is involved in the cortical cap protrusion, Ca^2+ ^was chelated by culture of the eggs in 50 μM BAPTA/AM (1,2-bis (aminophenoxy)-ethane-N, N, N', N'-tetraacetic acid) [13,15]. It was noted that the cortical cap protrusion was completely suppressed by BAPTA/AM (Figure 3F), suggesting that Ca^2+ ^is required for the cortical cap protrusion, most likely by activating myosin II contractility [16]. Consistently, inhibition of myosin II contraction by using blebbistatin [17] or myosin light chain kinase by ML-7 [18] all blocked the cortical cap protrusion (Figure 3G, H). These results suggest that the observed cortical cap protrusion is caused by Ca^2+ ^-mediated myosin II contraction.

Furthermore, it is noted that disruption of myosin II contractility either by blebbistatin or ML-7 [18] had no effect on chromosome segregation but blocked the spindle rotation as indicated by the spindle being positioned parallel to the cortex (Figure 3G, H), consistent with a previous report [19].

### Furrow induction by the spindle midzone in the oocyte is distance-dependent

As shown above, the membrane furrow induction by the anaphase spindle midzone was always observed at the adjacent cortex and never observed at the distant cortex (observation of over 130 activated eggs). To test whether the anaphase spindle midzone induces cortical membrane furrowing in a distance-dependent manner, DNA beads were injected into the center of the eggs to induce spindle formation farther away from the cortex (Figure 4A). After inducing anaphase by SrCl_2_, the DNA bead-spindle underwent metaphase-anaphase transition but no membrane furrow and cytokinesis were induced by the anaphase spindle midzone (Figure 4B, observation of 15 eggs). In contrast, when DNA beads were injected to the cortex which induced spindle formation close to the cortex (data not shown), the DNA bead-spindle induced cortical furrowing and polar body extrusion after egg activation (Figure 4C) [20]. These results suggest that the furrow induction by the anaphase spindle midzone in the oocytes is distance-dependent.

## Discussion

### Chromosomal determination of asymmetric meiotic division in the oocytes

In contrast to mitotic cells where cell polarity is specified by the spindle microtubules [21] or centrosomes [22], meiotic chromosomes plays an important role in establishing cortical polarity of the oocytes [6] which are devoid of centrosomes [23]. In addition, the chromosomes also play a key role in organizing bipolar spindle formation in the oocytes [24,25] which is independent of cortical polarity. Thus, chromosomes play a dual role in defining asymmetric meiotic divisions in the oocytes by: 1) specifying a cortical polarity; 2) inducing spindle formation. However, the chromosome-induced two events are independent from each other and disruption of either has no effect on the other [6]. The uncoupling of chromosome segregation and cytokinesis suggests a lack of a functional spindle-position checkpoint during female meiosis.

Successful induction of ectopic polar body extrusion by microinjection of DNA beads has provided a useful tool to study asymmetric meiotic division in the oocytes. It is interesting to note that while the very nature of the reductional chromosome segregations (a step-wise segregation between the homologous chromosomes and then between the sister chromatids) is determined by the special organization of the meiotic chromosomes [26], the spindle formation and polar body extrusion however, are induced by more general chromosome signals. This "inconsistency" of the chromosomal behavior during meiosis makes oocytes more vulnerable to generating errors during asymmetric meiotic divisions, given that any isolated chromosomal structure has a potential to induce an ectopic polar body extrusion in an oocyte [4,7].

It should be pointed out that although microtubules can self-assemble into a bipolar spindle in mouse oocytes in the absence of chromosomes [6,27], the formed chromatin-less spindle is unable to induce either cortical cap formation [6] or any cortical protrusion (unpublished observation). The lack of function of the chromatin-less spindle may ensure polar body extrusion induced only by the chromosome-induced spindle.

To reduce the chromosome number in half and preserve as much as possible the cytoplasmic components in the mature oocytes, the chromosome segregation must be coupled with polar body extrusion. This is achieved by chromosome migration and anchoring to the cortex during the process of the two meiotic divisions. Recent reports show that the chromosome cortical migration requires Formin-2-mediated actin assembly [28-32]. Our previous report shows that the chromosomes induce cortical polarization in a distance-dependent manner [6]. Through a close-range induction of cortical polarity and spindle formation by the chromosomes, the chromosome segregation by the spindle is spatially coupled to the actomyosin-driven cortical protrusion. After anaphase onset, the spindle midzone induces a unilateral furrow at the adjacent cortex but not the distal cortex (Figure 3A, B), which suggests that the midzone-induced furrow during meiosis is also distance-dependent. The distance-dependent furrow inducing activity by the anaphase spindle midzone in the oocytes requires a perpendicular position of the spindle relative to the protruding cortex, which may maximize the constriction of the contractile ring at the shoulder region of the cortical protrusion, leading to the final abscission of polar body.

### Coordination of the chromosome-induced cortical protrusion and the spindle midzone-induced cortical furrowing during polar body extrusion

**Figure 5:**
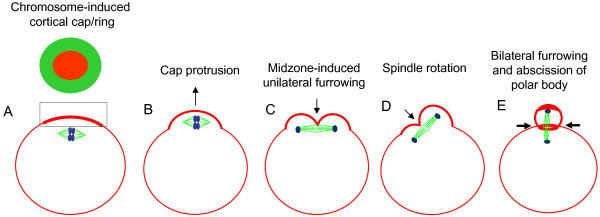
**A simplified model depicting the cortical cap protrusion and spindle midzone-induced membrane furrowing during polar body extrusion**. (A) Chromosomes induce formation of a cortical actomyosin cap/ring prior to polar body extrusion. The squared region of the cortical cap/ring is shown on the top, an actin cap (red) surrounded by a myosin II ring (green). (B) Egg activation induces the cortical cap protrusion. (C) The anaphase spindle midzone induces unilateral furrowing. (D) Spindle rotation. (E) Spindle midzone induces bilateral furrowing and abscission of polar body.

The chromosome-induced spindle and cortical actomyosin cap play distinct roles in polar body extrusion (Figure 5). Our results show that the cortical cap undergoes protrusion after anaphase onset which is important for spindle rotation during polar body extrusion. The significance of the cortical cap protrusion may be two fold: 1) it may generate an unbalanced force for spindle rotation and 2) it may coordinate with the spindle midzone-induced furrow to correctly position the anaphase spindle midzone to the neck region of the bulging cortex for efficient polar body abscission. It is interesting to note that in contrast to the first mitosis in the zygotes where a centrally positioned midzone induces robust bilateral furrowing from the center of the oocyte [33], the meiotic spindle can only induce a unilateral furrow from the closer cortex (Figure 3A, 5). This suggests that there is a significant difference between mitosis and meiosis with regard to the ability of the anaphase spindle midzone to induce cytokinetic furrows. It seems that the furrow-inducing ability by the spindle midzone is restricted in the meiotic oocytes so that it can only induce cortical furrowing within a very close distance. This distance-dependence, as well as that of induction of the cortical cap by the chromosomes, may provide a double checkpoint to ensure the highly asymmetric divisions in the oocytes.

## Conclusions

Chromosomes determine the site for polar body extrusion by inducing a cortical actomyosin cap/ring and a bipolar spindle. After anaphase onset, the cortical cap undergoes protrusion and the spindle midzone induces membrane furrowing in a distance-dependent manner. A coordination of the cortical cap protrusion with the distance-dependent membrane furrowing induced by the spindle midzone is critical for successful polar body extrusion.

## Methods

### Oocyte and egg collection and culture

All the experiments were performed following the animal protocol (protocol number 04638) that is approved by the IACUC at Harvard Medical School.

Female mice of CD1 at age of 4-6 week-old were superovulated by injection of pregnant mare serum gonadotropin (PMSG) and human chorionic gonadotropin (hCG) as described previously [34] and the ovulated eggs were collected from oviducts 14-15 h after hCG injection as previously described [11].

### Microinjection

DNA beads were prepared as described [6,25] and injected into the MII eggs as described previously [6].

Briefly, a cluster of 3-5 DNA beads was injected into the cortex distal to the maternal meiotic chromosome/spindle to induce ectopic formation of a cortical cap and a bipolar spindle as described previously [6,11] for convenient observation of the cortical response and ectopic polar body extrusion. Naked beads that are not coated with DNA were injected into the eggs as a control.

The injected eggs were cultured in M16 (Chemicon) at 37°C in an atmosphere of 5% CO_2 _in air for different periods of time to evaluate the cortical actomyosin cap and ring formation and spindle induction. To determine the time required for the cortical actin cap and spindle induction by DNA beads, the eggs were fixed every 20 min after microinjection to immunostain actomyosin and spindle microtubules as described before [6].

### Drug treatments

Eggs were treated with blebbistatin (Calbiochem, San Diego, CA, USA) at 100 μM to specifically inhibit myosin II contractility [6,35] for 30-40 min, ML-7 (Sigma) at 50 μM to inhibit myosin light chain kinase [18,19,36], and nocodazole (Sigma) at 10 μM for 30-40 min prior to bead injection. For Lat-A treatment, we injected eggs with DNA beads first and then transferred the bead-injected eggs to 100 μM Lat-A (Sigma) within 5 min to ensure an optimal survival of the injected eggs [6].

### Egg activation

Eggs were either parthenogenetically activated by 10 mM SrCl_2 _in Ca^2+ ^free CZB medium [37,38] or fertilized with sperm in vitro as described previously to induce anaphase onset [20,34]. For convenient observation of the chromosome-induced cortical polarization and polar body extrusion, zona pellucida was removed from the eggs using acidic Tyrode solution [34].

### Immunofluorescence and Confocal microscopy

Eggs were fixed, immunostained and mounted on slides as described previously [6,11,18]. The antibodies and the reagents used for immunostaining to visualize actin, myosin II, cortical granules, microtubules, DNA were described previously [6,11,18,20]. Spindle midzone was stained using rabbit survin antibody (Abcam, Cambridge, UK, 1:400) and Alexa 633 conjugated secondary antibody. All the images were acquired by using a 40× or a 63× oil objective on a Zeiss LSM510 Confocal microscope. To construct 3D images, a stack of at least 50 Z-section images spanning all the observed structures was collected and reconstructed using Zeiss LSM-CFS. The images were processed using Photoshop 7.0 and assembled in Canvas 11.

### Live imaging of the oocytes during polar body extrusion

To visualize spindle and chromosome behavior during DNA bead-induced ectopic polar body extrusion, DNA bead-injected eggs were injected with rhodamine-labeled tubulin (Cytoskeleton, Denver), at a concentration of 2 mg/ml (injection volume 5 pl) and stained with Hoechst 33342 (Sigma) at a concentration of 5 ng/ml in a chamber containing Ca^2+ ^free CZB. The eggs were activated with SrCl_2 _as described above and observed using a Zeiss 510 NLO Confocal microscopy to visualize the rhodamine-labeled spindle and Hoechst-labeled DNA. Live imaging was performed on an on- stage incubator which maintains temperature at 37°C and 5% CO2. Z-section images were collected at a time interval of 7 min.

## Competing interests

The authors declare that they have no competing interests.

## Authors' contributions

All authors have read and approved the final manuscript. MD designed the experiments and QW and MD performed all the experiments and data analysis. MD and CR wrote the manuscript and MD is responsible for the scientific contents of the manuscript.

## Supplementary Material

Additional file 1 **A 4D movie showing the DNA bead-induced ectopic polar body extrusion during meiosis II**. The DNA beads (shown in blue) were injected at 10 O'clock position. Microtubules were labeled by microinjection of rhodamine conjugated tubulin. Note that the DNA bead spindle underwent rotation from horizontal to vertical position and the DNA beads were kept in the eggs after ectopic polar body extrusion. Maternal chromosomes are out of the z-sections. Click here for file

## References

[B1] Cowan CR Hyman AA Asymmetric cell division in C. elegans: cortical polarity and spindle positioning Annu Rev Cell Dev Biol 2004 20 427 453 10.1146/annurev.cellbio.19.111301.113823 15473847

[B2] Siller KH Doe CQ Spindle orientation during asymmetric cell division Nat Cell Biol 2009 11 365 374 10.1038/ncb0409-365 19337318

[B3] Gonczy P Mechanisms of asymmetric cell division: flies and worms pave the way Nat Rev Mol Cell Biol 2008 9 355 366 10.1038/nrm2388 18431399

[B4] Maro B Johnson MH Webb M Flach G Mechanism of polar body formation in the mouse oocyte: an interaction between the chromosomes, the cytoskeleton and the plasma membrane J Embryol Exp Morphol 1986 92 11 32 3723057

[B5] Van Blerkom J Bell H Regulation of development in the fully grown mouse oocyte: chromosome-mediated temporal and spatial differentiation of the cytoplasm and plasma membrane J Embryol Exp Morphol 1986 93 213 238 3734683

[B6] Deng M Suraneni P Schultz RM Li R The Ran GTPase Mediates Chromatin Signaling to Control Cortical Polarity during Polar Body Extrusion in Mouse Oocytes Dev Cell 2007 12 301 308 10.1016/j.devcel.2006.11.008 17276346

[B7] Deng M Li R Sperm chromatin-induced ectopic polar body extrusion in mouse eggs after ICSI and delayed egg activation PLoS One 2009 4 e7171 10.1371/journal.pone.0007171 19787051 PMC2746308

[B8] Simerly C Nowak G de Lanerolle P Schatten G Differential expression and functions of cortical myosin IIA and IIB isotypes during meiotic maturation, fertilization, and mitosis in mouse oocytes and embryos Mol Biol Cell 1998 9 2509 2525 9725909 10.1091/mbc.9.9.2509 PMC25518

[B9] Glotzer M Cleavage furrow positioning J Cell Biol 2004 164 347 351 10.1083/jcb.200310112 14757750 PMC2172245

[B10] von Dassow G Concurrent cues for cytokinetic furrow induction in animal cells Trends Cell Biol 2009 19 165 173 10.1016/j.tcb.2009.01.008 19285868

[B11] Deng M Kishikawa H Yanagimachi R Kopf GS Schultz RM Williams CJ Chromatin-mediated cortical granule redistribution is responsible for the formation of the cortical granule-free domain in mouse eggs Dev Biol 2003 257 166 176 10.1016/S0012-1606(03)00045-9 12710965

[B12] Brunet S Maro B Cytoskeleton and cell cycle control during meiotic maturation of the mouse oocyte: integrating time and space Reproduction 2005 130 801 811 10.1530/rep.1.00364 16322540

[B13] Kline D Kline JT Repetitive calcium transients and the role of calcium in exocytosis and cell cycle activation in the mouse egg Dev Biol 1992 149 80 89 10.1016/0012-1606(92)90265-I 1728596

[B14] Tomashov-Matar R Tchetchik D Eldar A Kaplan-Kraicer R Oron Y Shalgi R Strontium-induced rat egg activation Reproduction 2005 130 467 474 10.1530/rep.1.00746 16183864

[B15] Tsien RY New calcium indicators and buffers with high selectivity against magnesium and protons: design, synthesis, and properties of prototype structures Biochemistry 1980 19 2396 2404 10.1021/bi00552a018 6770893

[B16] Somlyo AP Somlyo AV Signal transduction and regulation in smooth muscle Nature 1994 372 231 236 10.1038/372231a0 7969467

[B17] Straight AF Cheung A Limouze J Chen I Westwood NJ Sellers JR Mitchison TJ Dissecting temporal and spatial control of cytokinesis with a myosin II Inhibitor Science 2003 299 1743 1747 10.1126/science.1081412 12637748

[B18] Deng M Williams CJ Schultz RM Role of MAP kinase and myosin light chain kinase in chromosome-induced development of mouse egg polarity Dev Biol 2005 278 358 366 10.1016/j.ydbio.2004.11.013 15680356

[B19] Matson S Markoulaki S Ducibella T Antagonists of myosin light chain kinase and of myosin ii inhibit specific events of egg activation in fertilized mouse eggs Biol Reprod 2006 74 169 176 16207836 10.1095/biolreprod.105.046409

[B20] Deng M Gao J Suraneni P Li R Kinetochore-independent chromosome poleward movement during anaphase of meiosis II in mouse eggs PLoS ONE 2009 4 e5249 10.1371/journal.pone.0005249 19365562 PMC2664963

[B21] Siegrist SE Doe CQ Microtubule-induced cortical cell polarity Genes Dev 2007 21 483 496 10.1101/gad.1511207 17344411

[B22] de Anda FC Pollarolo G Da Silva JS Camoletto PG Feiguin F Dotti CG Centrosome localization determines neuronal polarity Nature 2005 436 704 708 10.1038/nature03811 16079847

[B23] Szollosi D Calarco P Donahue RP Absence of centrioles in the first and second meiotic spindles of mouse oocytes J Cell Sci 1972 11 521 541 5076360 10.1242/jcs.11.2.521

[B24] Caudron M Bunt G Bastiaens P Karsenti E Spatial coordination of spindle assembly by chromosome-mediated signaling gradients Science 2005 309 1373 1376 10.1126/science.1115964 16123300

[B25] Heald R Tournebize R Blank T Sandaltzopoulos R Becker P Hyman A Karsenti E Self-organization of microtubules into bipolar spindles around artificial chromosomes in Xenopus egg extracts Nature 1996 382 420 425 10.1038/382420a0 8684481

[B26] Paliulis LV Nicklas RB The reduction of chromosome number in meiosis is determined by properties built into the chromosomes J Cell Biol 2000 150 1223 1232 10.1083/jcb.150.6.1223 10995430 PMC2150703

[B27] Brunet S Polanski Z Verlhac MH Kubiak JZ Maro B Bipolar meiotic spindle formation without chromatin Curr Biol 1998 8 1231 1234 10.1016/S0960-9822(07)00516-7 9811610

[B28] Leader B Lim H Carabatsos MJ Harrington A Ecsedy J Pellman D Maas R Leder P Formin-2, polyploidy, hypofertility and positioning of the meiotic spindle in mouse oocytes Nat Cell Biol 2002 4 921 928 10.1038/ncb880 12447394

[B29] Dumont J Million K Sunderland K Rassinier P Lim H Leader B Verlhac MH Formin-2 is required for spindle migration and for the late steps of cytokinesis in mouse oocytes Dev Biol 2007 301 254 265 10.1016/j.ydbio.2006.08.044 16989804

[B30] Li H Guo F Rubinstein B Li R Actin-driven chromosomal motility leads to symmetry breaking in mammalian meiotic oocytes Nat Cell Biol 2008 10 1301 1308 10.1038/ncb1788 18836438

[B31] Azoury J Lee KW Georget V Rassinier P Leader B Verlhac MH Spindle positioning in mouse oocytes relies on a dynamic meshwork of actin filaments Curr Biol 2008 18 1514 1519 10.1016/j.cub.2008.08.044 18848445

[B32] Schuh M Ellenberg J A new model for asymmetric spindle positioning in mouse oocytes Curr Biol 2008 18 1986 1992 10.1016/j.cub.2008.11.022 19062278

[B33] Hiiragi T Solter D First cleavage plane of the mouse egg is not predetermined but defined by the topology of the two apposing pronuclei Nature 2004 430 360 364 10.1038/nature02595 15254539

[B34] Hogan B Costantini F Lacy E Manipulating the mouse embryo: a laboratory manual 1986 Cold Spring Harbor, N.Y.: Cold Spring Harbor Laboratory

[B35] Straight AF Cheung A Limouze J Chen I Westwood NJ Sellers JR Mitchison TJ Dissecting temporal and spatial control of cytokinesis with a myosin II Inhibitor Science 2003 299 1743 1747 10.1126/science.1081412 12637748

[B36] Saitoh M Ishikawa T Matsushima S Naka M Hidaka H Selective inhibition of catalytic activity of smooth muscle myosin light chain kinase J Biol Chem 1987 262 7796 7801 3108259

[B37] Chatot CL Ziomek CA Bavister BD Lewis JL Torres I An improved culture medium supports development of random-bred 1-cell mouse embryos in vitro J Reprod Fertil 1989 86 679 688 10.1530/jrf.0.0860679 2760894

[B38] Kishigami S Wakayama S Thuan NV Ohta H Mizutani E Hikichi T Bui HT Balbach S Ogura A Boiani M Wakayama T Production of cloned mice by somatic cell nuclear transfer Nat Protoc 2006 1 125 138 10.1038/nprot.2006.21 17406224

